# Supplementary material for: The Positive Impact and Associated Mechanisms of Physical Activity on Mental Health in Underprivileged Children and Adolescents: An Integrative Review
Source: Behav Sci (Basel). 2020 Nov 8;10(11):171. doi: 10.3390/bs10110171 (PMC7695200; doi:10.3390/bs10110171)
Supplement: Supplementary file 1 [file behavsci-10-00171-s001.pdf]

Table S1. The Demographics, Design and Quality Assessment for included studies.

Study	Methodology, Aim, Eligibility	Participants, Sampling and Setting	Key intervention details	Data collection methods, focus and analysis	Quality assessment	Strength of evidence*
1.Ullrich-French, McDonough and Smith [30] Social connection and psychological outcomes in a physical activity-based youth development setting.	*Methodology*: Intervention *Aim*: To see if a change in perceived social connectedness, through a PA program, leads to a positive change in psychological outcomes. *Eligibility Criteria*: Low SES, could read and speak English, were able to complete both questionnaires.	*Participants*: 197 low SES, children aged between 9 and 16 years (95 boys, 102 girls) *Sampling Technique*: National Youth sports programme participants (have to be low SES to attend), all attendees were invited to participate *Setting*: University-based summer camp *Country*: USA	*Type of physical activity/sport:* A range of sports, swimming, walking, recreational games *Duration:* 8am-2pm (70% of the day devoted to PA) *Frequency*: Daily for 4 weeks *Competitive or Non-Competitive:* Non-competitive, focused on personal improvement and lifestyle activities *Intensity:* Not stated *Group or Individual*: Group – children were assigned to groups of 10-12 individuals of a similar age *Control group:* No *Adult involvement:* Each group was assigned an adult leader/mentor who stayed with them throughout. Specialist instructors taught skill improvement in each sport. *Mental health outcome measured:* Social competence, physical competence, global self-worth, social self-worth	*Methods*: Pre and post programme questionnaires *Method for measuring mental health outcome:* Social and Physical Competence and Global Self-worth - Harter's (1985) self- perception profile for children [72] Physical Self-worth - Whitehead's (1995) children's physical self-perception profile [73] *Non-Mental Health Outcomes Measured and Methods:* Leader Support – Adapted teacher support subscale [73] Attraction to Physical Activity – Adapted version of Brustad's (1993) [75] Children's Attraction to Physical Activity Scale [76] Hope – Children's Hope Scale [77] *Analysis*: Quantitative	5/5	Level II evidence - Good Quality Cohort Study
2. Breslin et al. [29] Physical activity and wellbeing of 8–9-year-old children from	*Methodology*: Cross-sectional study, part of a	*Participants*: 673 Children aged 8-9 years (347 boys and 326 girls), low SES	*Type of physical activity/sport:* Moderate to vigorous PA	*Methods*: Questionnaires and Surveys. PA measured using the	3/5	Level IV Evidence - Poor Quality

social disadvantage: An all-Ireland approach to health	larger longitudinal, cluster randomised control trial<br>*Aim:* To see if 60 minutes of moderate to vigorous PA daily enhances wellbeing<br>*Eligibility Criteria*: primary schools in socially disadvantaged areas included	*Sampling Technique*: Schools serving areas of deprivation were invited to participate.<br>*Setting*: Primary schools<br>*Country*: Ireland	*Duration:* 60 minutes<br>*Frequency*: Daily<br>*Competitive or Non-competitive:* N/A<br>*Intensity:* moderate to vigorous<br>*Group or Individual*: Not stated<br>*Control group:* No<br>*Adult involvement:* Questionnaire was administered by research team members. PA may or may not have involved adult supervision or instruction<br>*Mental health outcome measured:* Psychological wellbeing, social support	Health Behaviour in School-Aged children survey.<br>*Method for measuring mental health outcome:* Kidscreen-27 questionnaire [48]<br>*Non-Mental Health Outcomes Measured and Methods:* Physical wellbeing – Kidscreen-27 Questionnaire [48]<br>*Analysis*: Quantitative		Cross-sectional study
3. Rothon et al. [63]<br>Physical activity and depressive symptoms in adolescents: a prospective study	*Methodology*: cross-sectional and longitudinal prospective cohort study. Follow up was conducted 2 years later.<br>*Aim*: To examine the relationship between physical activity and depressive symptoms.<br>*Eligibility Criteria*: All year 7 and year 9 pupils in selected schools were eligible to participate	*Participants*: 2093 adolescents (1025 boys, 1068 girls) aged 11-14 from schools in deprived areas of East London<br>*Sampling Technique*: 30 schools randomly selected which serve deprived areas<br>*Setting*: Schools<br>*Country*: UK	*Type of physical activity/sport:* not specified<br>*Duration:* Variable<br>*Frequency*: Variable<br>*Competitive or Non-Competitive:* Not stated<br>*Intensity:* Moderate to vigorous<br>*Group or Individual*: not specified<br>*Control group:* No<br>*Adult involvement:* Researchers administered the questionnaire and took physical measurements.<br>*Mental health outcome measured:* Depression	*Methods*: Data was collected from the 'Research with East London Adolescents: Community Health Survey' (RELACHS)<br>*Method for measuring mental health outcome:* Short moods and feelings questionnaire (SMFQ) [78]<br>*Analysis*: Quantitative	2/5	Level III Evidence - Poor Quality Cohort study
4. Sethi et al. [64]<br>Yoga improves attention and self-esteem in underprivileged girl student	*Methodology*: Intervention<br>*Aim*: To see if a yoga intervention improves self-esteem	*Participants*: 60 low-income adolescent girls aged 14-17	*Type of physical activity/sport:* Yoga<br>*Duration:* 3 hours	*Methods*: Questionnaire completed pre- and post-test	4/5	Level II Evidence - Good Quality

	Eligibility Criteria: Healthy girls with no illness or disability	*Sampling Technique*: Not stated<br>*Setting*: a single school<br>*Country*: India	*Frequency*: Daily for 5 days<br>*Competitive or Non-Competitive:* Non-competitive<br>*Intensity:* N/A<br>*Group or Individual*: Group<br>*Adult involvement:* Adults taught the integrated yoga module. Data was collected by an individual unconnected to the research, with the guidance of a psychologist<br>*Control group:* No<br>*Mental health outcome measured:* Self-esteem	*Method for measuring mental health outcome:* Rosenberg self-esteem scale [79]<br>*Analysis*: Quantitative		Cohort Study
5. Shachar et al. [65]<br>Reducing child aggression through sports intervention: The role of self-control skills and emotions	*Methodology*: Non-randomised control trial/ intervention, quasi-experimental<br>*Aim*: To use a sport intervention to reduce aggressive behaviours and enhance self-control in young children<br>*Eligibility Criteria*: child demonstrated aggressive behaviour; self-declared liking sports; received no treatment for psychological issues	*Participants*: 649 children (492 boys, 157 girls) from underprivileged areas<br>*Sampling Technique*: 39 Schools selected by research team and 21 selected to receive intervention, 18 selected to be control – teaching staff selected pupils<br>*Setting*: After school activity<br>*Country*: Israel	*Type of physical activity/sport:*2 hours of martial arts/ 3 hours of group sports<br>*Duration:*5 hours over a week<br>*Frequency*: weekly for 24 weeks<br>*Competitive or Non-Competitive:* Not stated<br>*Intensity:* Not stated<br>*Group or Individual*: Group<br>*Control group:* Yes - waitlisted<br>*Adult involvement:* Specialist coaches delivered the sessions and were monitored by a member of school staff<br>*Mental health outcome measured:* Aggression, self-control, emotional regulation	*Methods*: Questionnaires completed pre- and post-test<br>*Method for Measuring mental health outcome:* Aggression Questionnaire [80]; Self-control skills scale [81]; Positive and Negative Affect Schedule [82]<br>*Analysis*: Quantitative	4/5	Level II Evidence - Good Quality Cohort Study

6. Bonhauser et al. [62] Improving physical fitness and emotional well-being in adolescents of low socioeconomic status in Chile: results of a school-based controlled trial	*Methodology*: Quasi-experimental design *Aim*: To use a PE intervention to improve students' self-esteem *Eligibility Criteria*: children aged 15 years old in the 9th grade of a school in a low SES area of Chile participated	*Participants*: 198 adolescents aged 15 years (96 boys, 102 girls) *Sampling Technique*: 2 classes were randomly selected for the intervention and 2 for control from one school *Setting*: School *Country*: Chile	*Type of physical activity/sport:* stretching, walking, running jumping, athletics, volleyball, dance, aerobics, soccer, basketball *Duration:* 90 minutes *Frequency*: 3 times a week, for 40 weeks *Competitive or Non-competitive:* Not stated *Intensity:* variable but progressively increasing throughout each session *Group or Individual*: group *Control group:* Yes – Students did one 90 min standard PE lesson weekly *Adult involvement:* Sessions taught by 2 instructors, intervention planned by teachers and students *Mental health outcomes measured:* Self-esteem, anxiety, and depression	*Methods*: Questionnaires *Method for Measuring mental health outcome:* Anxiety and Depression - Hospital Anxiety Depression Scale [83] Self-esteem - Tennessee Self-concept scale [84] *Analysis*: Quantitative	5/5	Level II Evidence - Good Quality Cohort Study
7. Beaulac et al. [36] 'Bigger than hip-hop?' Impact of a community-based physical activity program on youth living in a disadvantaged neighbourhood in Canada	*Methodology*: Intervention *Aim*: To explore if a hip-hop dance program improves wellbeing in disadvantaged adolescents *Eligibility Criteria*: Live in low SES area of Ottawa, able to take part in moderate to high intensity exercise, aged between 11 and 16 years, spoke English	*Participants*: 67 low SES adolescents aged 11 to 16 (12 boys, 55 girls); 4 personnel; 13 parents *Sampling Technique*: Advertised in schools, shopping centre and community events – all youth who applied within this low SES area were invited to participate *Setting*: community centre	*Type of physical activity/sport:* Hip-Hop Dance *Duration:*1hr 15 mins *Frequency*: weekly for 13 weeks *Competitive or Non-Competitive:* Non-Competitive *Intensity:* Moderate-high *Group or Individual*: Group - 2 separate sessions offered: girls only and co-ed	*Methods*: Questionnaire, interviews, focus groups, survey *Method for Measuring mental health outcome:* Interviews *Analysis*: Qualitative	3/9	Moderate – serious concerns

		Country: Canada	*Control Group:* No<br>*Adult involvement:* 2 adult specialist dance instructors, one black female, one white male.<br>*Mental health outcome measured:* Psychological wellbeing, social skills, and peer attachments<br>*Cost to participants:* Free			
8. Riley and Anderson-Butcher [37]<br>Participation in a summer sport-based youth development program for disadvantaged youth: Getting the parent perspective	*Methodology*: Grounded Theory<br>*Aim*: To explore the impact of a sports program designed for positive youth development on low income children<br>*Eligibility Criteria*: low-income families, child was aged 10-12 during 2009 camp and attended again in 2010	*Participants*: 10 parents with children aged 11-13 who were participating for the second year<br>*Sampling Technique*: Randomly selected from all eligible participants<br>*Setting*: summer camp on university campus<br>*Country*: USA	*Type of physical activity/sport:* Sport-based activities involving self-control, effort, teamwork, social responsibility<br>*Duration:* 8am to 2pm<br>*Frequency*: Daily for 19 days<br>*Competitive or Non-Competitive:* Non-competitive<br>*Intensity:* Not stated<br>*Group or Individual*: Group<br>*Control group:* No<br>*Adult involvement:* Each group has a counsellor<br>*Mental health outcome measured:* social competence, social skills, connectedness, self-control, effort, teamwork<br>*Cost to participants:* Free	*Methods*: Semi-structured interviews with parents<br>*Method for Measuring mental health outcome:* Interviews with parents<br>*Analysis*: Qualitative	9/9	Very Minor Concerns
9. Crews, D.J., Lochbaum, M.R., and Landers, D.M. [61]<br>Aerobic Physical Activity Effects on Psychological Well-Being in Low-Income Hispanic Children	*Methodology*: Intervention<br>*Aim*: To see if an aerobic exercise program enhanced psychological wellbeing<br>*Eligibility Criteria*: Low income, Hispanic and in grade 4 of schools in local area	*Participants*: 66 low-income Hispanic students in grade 4 (33 boys, 33 girls)<br>*Sampling Technique*: randomly assigned to aerobic exercise or control group<br>*Setting*: School	*Type of physical activity/sport:* Aerobic Exercise<br>*Duration:* 20 minutes<br>*Frequency*: 3 times per week, for 6 weeks<br>*Competitive or Non-Competitive:* Non-competitive	*Methods*: Questionnaires<br>*Method for Measuring mental health outcome:* Trait anxiety inventory for children [85]; Beck depression inventory II [86] and Rosenberg self-esteem scale [79]<br>*Analysis*: Quantitative	2/5	Level II Evidence - Poor Quality RCT

		Country: USA	*Intensity:* High intensity<br>*Group or Individual*: Group<br>*Control group:* Yes – same duration and frequency but lower, controlled intensity<br>*Adult involvement:* sessions run by trained adults<br>*Mental health outcome measured:* Anxiety, Depression, self-esteem			
10. Terry, Hahn and Zimjanovic [31]<br>Effects of a sport programme (Box'Tag®) on disadvantaged youth participants	*Methodology*: Randomised Control Trial Intervention<br>*Aim*: To assess the effects of a novel sports intervention, Box`Tag on physical fitness and psychological wellbeing of disadvantaged children<br>*Eligibility Criteria*: All children in grade 7 of a school serving low-income families were invited to participate.	*Participants*: 51 disadvantaged 11 and 12-year-old children (28 boys, 23 girls)<br>*Sampling Technique*: children volunteered and were randomly assigned to either the intervention or control group<br>*Setting*: School during lunch break<br>*Country*: Australia	*Type of physical activity/sport:* Box'Tag (low risk boxing)<br>*Duration:* 35 minutes<br>*Frequency*: 2 to 3 times per week for 8 weeks.<br>*Competitive or Non-Competitive:* Competitive<br>*Intensity:* High<br>*Group or Individual*: Group<br>*Control group:* Yes – Largely non-physical social skills session 'Rock and Water'<br>*Adult involvement:* Sessions were run by trained adults<br>*Mental health outcome measured:* various psychological and emotional wellbeing factors	*Methods*: Questionnaires<br>*Method for Measuring mental health outcome:* Strengths and Difficulties Questionnaire [87] and Brunel Mood Scale [88], Physical activity questionnaire for older children [89]<br>*Analysis*: Quantitative	3/5	Level II Evidence - Poor Quality RCT
11. Anderson-Butcher et al. [90]<br>Maximising Youth Experiences in Community Sport Settings: The Design and Impact of the LiFE Sports Camp	*Paradigmatic position*: Research is a specific objective within the camp, and researchers worked as leaders, researchers' positionality may therefore influence the results.	*Participants*: 287 disadvantaged youth aged 9-16 (169 boys, 118 girls)<br>*Sampling Technique*: Youth were invited to participate in the camp.	*Type of physical activity/sport:* Basketball, American football, soccer, lacrosse, health and fitness, social dance, softball, and swimming<br>*Duration:*19 days	*Methods*: Pre- and Post-test surveys.<br>*Method for Measuring mental health outcome:* Modified Perceived Social Competence Scale [91]; Social Sports Experience Scale [92]; Athletic Competence Scale [93]; Multidimensional Sportspersonship	4/5	Level III Evidence - Moderate Quality Cohort Study

	Methodology: Intervention<br>*Aim*: To explore the impact of a community sports program on the development of social and sports skills among vulnerable youth<br>*Eligibility Criteria*: Disadvantaged youth, attended for at least 15/19 days, reported being honest in completing the survey.	Parents and youth 14+ were asked for permission to take part in the study<br>*Setting*: Learning in Fitness and Education (LiFE) Sports camp on university campus, Ohio<br>*Country*: USA	*Frequency*: 1 hour of play-based social skill instruction daily; 3 1-hour sport coaching sessions that incorporate social skills daily.<br>*Competitive or Non-Competitive:* Both<br>*Intensity:* Moderate to high<br>*Group or Individual*: Group<br>*Control group:* No<br>*Adult involvement:* Trained research assistants were available to answer questions from the children completing the surveys. Researchers were group leaders.<br>*Mental health outcomes measured:* social competence in sport; sport competence; self-control; effort; teamwork; social responsibility and belonging	Orientations Scale [94]; The Teamwork Scale [95]; Social responsibility scale [92] and Belonging scale [95].<br>*Analysis*: Quantitative		
12. Salvini et al. [38]<br>Physical activity and health-related quality of life among schoolchildren from disadvantaged neighbourhoods in Port Elizabeth, South Africa	*Methodology*: Cross-sectional study<br>*Aim*: To determine if higher levels of PA and cardio-respiratory fitness lead to greater Health-related quality of life (HRQoL) in disadvantaged children<br>*Eligibility Criteria*: All children in grade 4 of eight schools serving disadvantaged	*Participants*: 832 disadvantaged children aged 8-12 years (415 boys, 417 girls)<br>*Sampling Technique*: All children in grade 4 of selected schools were invited to participate<br>*Setting*: School<br>*Country*: South Africa	*Type of physical activity/sport:* Not stated<br>*Duration:* N/A<br>*Frequency*: varied<br>*Competitive or Non-Competitive:* Not stated<br>*Intensity:* Not stated<br>*Group or Individual*: Not stated<br>*Control group:* No<br>*Adult involvement:* Tests were supervised by a teacher and a trained research assistant.	*Methods*: Questionnaire + running endurance test<br>*Method for Measuring mental health outcome:* KIDSCREEN-27 [48]<br>*Analysis*: Quantitative	5/5	Level III Evidence - Good Quality Cross-sectional Study

	communities in Port Elizabeth were eligible		*Mental health outcome measured:* HRQoL Psychological wellbeing			
13. Lubans et al. [35] Mediators of Psychological well-being in adolescent boys	*Methodology*: Cluster Randomised control trial (ATLAS program intervention) *Aim*: To explore the effect of the ATLAS intervention on psychological wellbeing of adolescent boys and its mediating mechanisms. *Eligibility Criteria*: Adolescents in grade 7 of schools serving deprived areas, who self-reported failing to meet PA and leisure-time screen usage guidelines	*Participants*: 361 low SES adolescent boys, mean age 12.7 *Sampling Technique*: Randomised at school level *Setting*: Schools *Country*: Australia	*Type of physical activity/sport:* Sport session focussed on improving muscular fitness *Duration:* 90 minutes *Frequency*: 1 session weekly for 20 weeks plus measurement of lifestyle PA *Competitive or Non-Competitive:* Not stated *Intensity:* Moderate to Vigorous *Group or Individual*: Group *Control group:* Yes – Waitlisted *Adult involvement:* Sport session delivered by teachers *Mental health outcome measured:* Psychological wellbeing	*Methods*: Questionnaires *Method for Measuring mental health outcome:* 8-item Flourishing Scale [95] *Analysis*: Quantitative	4/5	Level II Evidence - Moderate Quality RCT
14. Velásquez et al. [57] Yoga for the prevention of depression, anxiety, and aggression and the promotion of socioemotional competencies in school-aged children	*Methodology*: Intervention *Aim*: To see if a yoga programme can prevent anxiety, depression, and aggression in low SES children. *Eligibility Criteria*: All children in grades 5, 8 and 9 in selected school were invited to participate.	*Participants*: 125 children from grades 5, 8 and 9 (12-15 yrs). *Sampling Technique*: Random assignment to intervention or control *Setting*: Low SES status School *Country*: Colombia	*Type of physical activity/sport:* Yoga *Duration:* 2 hrs *Frequency*: Twice weekly for 12 weeks in an afterschool club *Competitive or Non-Competitive:* Non-competitive *Intensity:* Not stated *Group or Individual*: Group *Control group:* Yes - Waitlisted *Adult involvement:* Trained yoga	*Methods*: Pre- and post-test Questionnaires; peer assessments; focus groups *Method for Measuring mental health outcome:* Anxiety and Depression - Strengths and Difficulties Questionnaire [87]; Aggression -Peer Assessments; Empathy and Anger Management - Self-report Questionnaires *Analysis*: Mixed methods	2/5	Level II Evidence - Poor Quality RCT

			professionals ran the sessions *Mental health outcome measured:* Anxiety, depression, aggression *Cost to participants:* Free			
15. Frank et al. [32] Effectiveness of a School-Based Yoga Program on Adolescent Mental Health and School Performance: Findings from a Randomized Controlled Trial	*Methodology:* Randomised control trial Intervention *Aim:* To assess the effect of 'Transformative Life Skills', a yoga-based socio-emotional wellness program on adolescent emotional regulation, prosocial behaviour and school function *Eligibility Criteria:* All students in grade 6 and 9 of the selected school could participate.	*Participants:* 159 adolescents (85 boys, 74 girls, from an inner-city low SES school *Sampling technique:* Students were randomly assigned to intervention or control groups *Setting:* School, during normal lesson time *Country:* USA	*Type of physical activity/sport:* Yoga *Duration:* 30 minutes *Frequency:*3 or 4 times a week for a school semester *Competitive or Non-Competitive:* *Intensity:* Not stated *Group or Individual:* Group Control group: Yes – same duration and frequency, alternative PA *Adult involvement:* Intervention was delivered by trained yoga professionals *Mental health outcome measured:* Stress, emotional regulation; aggression, positive and negative affect *Cost to participants:* Free	*Methods:* Questionnaires *Methods for measuring mental health outcome:* Attitudes towards violence scale [96]; Positive and Negative Affect Schedule for children [97]; Responses to Stress Questionnaire [98]; School engagement scale [99] *Analysis:* Quantitative	3/5	Level II Evidence - Poor Quality RCT
16. Berger et al. 672] Effects of Yoga on Inner-City Children's Wellbeing – A Pilot Study	*Methodology:* Randomised control trial intervention *Aim:* To examine the effects of a yoga intervention on children's wellbeing *Eligibility Criteria:* All children in the 4th and 5th grades of the 2 selected schools were entitled to participate. Both schools	*Participants:* 68 (24 boys, 47 girls) 4th and 5th grade students (9-11yrs) from 2 inner-city schools in the Bronx, New York *Sampling technique:* The children in one school were assigned to the yoga intervention, the children in the other school were the control group	*Type of physical activity/sport:* Yoga *Duration:* 1 hr *Frequency:* Weekly for 12 weeks *Competitive or Non-Competitive:* Non-competitive *Intensity:* Not stated *Group or Individual:* Group	*Methods:* Pre- and Post-test Questionnaires, post-test survey *Methods for measuring mental health outcome:* Harter's Self-Perception Profile for Children [72]; Perceptions of Physical Health Scale [67]; Yoga Teachings Scale [67]; Effects of Yoga on Well-being Survey [67] *Analysis:* Mixed methods	2/5	Level II Evidence - Poor Quality Cohort Study

	served a low SES population	*Setting:* Inner-city elementary schools, New York after school program<br>*Country:* USA	*Control group:* Yes – normal school curriculum<br>*Adult involvement:* One yoga teacher and one after-school club staff member ran the group<br>*Mental health outcome measured:* Global Self-worth, self-concept, emotional regulation<br>*Cost to participants:* Free		

Note * = levels of evidence are taken from Anon. [100].

3.3. Quality Assessment Summary

The full between and within study quality analysis can be found within the supplementary file.

3.3.1. Between Study Analysis

The quantitative randomised control trials were all poor to moderate quality studies with Crews et al. [61] scoring the lowest with 2/5 and Lubans et al. [35] scoring the highest with 4/5. The quality of the quantitative non-randomised studies ranged from poor to good, with Rothon et al. [63] scoring the lowest with 2/5 and three studies, Bonhauser et al. [62], Ullrich-French, McDonough and Smith [30] and Salvini et al. [38] scoring 5/5. Of the two included qualitative studies, Beaulac et al. [36] was a poor-quality study with 3/9, whereas Riley and Anderson-Butcher [37] was a high-quality study and scored the maximum 9/9. Both the mixed methods studies, Berger et al. [67] and Velásquez et al. [57] were low-quality with a score of 2/5. Overall, 4/16 studies scored 100% and 8/16 scored 80% or higher.

3.3.2. Within Tool Analysis

There was some consistency with the quality assessment of the quantitative RCTs, in that 4/4 studies provided complete outcome data and within 3/4 studies the participants adhered to the intervention throughout. Group comparability at baseline was achieved in 3/4 studies. However, a significant issue with the RCTs was that 0/4 studies provided evidence that the outcome assessors were blinded to the intervention.

In every included quantitative non-randomised study (8/8) the sample was representative of the target population and in 7/8 the intervention was administered as planned. The least consistent measure related to confounders with only 5/8 studies showing the confounders had been accounted for in the design and analysis of the study.

The two mixed methods studies [57,67] scored consistently throughout with both providing an adequate rationale for using mixed methods whilst also explaining and accounting for inconsistencies between qualitative and quantitative findings. However, they both failed to integrate the findings and the quality of both the qualitative and quantitative components was low.

There was a significant disparity in the quality assessment of the two included qualitative studies, with one regarded as high quality, 9/9 [37] and the other, poor quality, 3/9 [36]. There were three items which scored highly in both, the appropriateness of the qualitative methods, the derivation of findings from the data and the interpretation of the results in relation to the data.

Table S2. – Results for internal mental health and wellbeing outcomes.

Study	Psychological wellbeing (PW)	Self-worth (SW)	Self-esteem	Motivation towards physical activity	Hope	Depression	Anxiety	Emotional regulation	Other	*Any Identifiable biopsychosocial mechanisms and influencing factors*
**[30]**		**Physical self-worth (PSW) - significant increase**, F=11.12, η^2=0.05, *p*<0.01 - ***small effect*** **Global self-worth (GSW)- significant increase**, F=17.12, η^2 = 0.15 *p*<0.01 - ***moderate effect*** *T1 - Moderate +correlation with PSW (r=0.66) and Physical competence (PC) (r=0.48) T2 - Moderate + correlation with APA (r=0.64) and hope (r=0.44)*		**Attraction to PA (APA) No significant change** - Age was a moderator - ***12-16 yrs significant increase*** F=7.46, η^2 = 0.07, *p*<0.01 No change for 9-11 yrs **-** ***small effect*** T1 - Moderate +correlation with SW (r=0.53), PSW (r=0.64), PC (r=0.68) and SC (r=0.49) T2 - Moderate + correlation with hope (r=0.49)	**Hope - No significant change** T1 - Moderate +correlation with APA (r=0.47), PSW (r=0.45) and LS (r=0.43).					**Age was a moderator** – Leader Support (LS): 9-11 yrs, significant decrease but no change for 12-16 yrs; PSW and APA: 12-16 yrs significant increase, no change for 9-11 yrs **Gender was a moderator** – Social Competence (SC): only girls showed a significant increase. *Increase in PC predicted increase in PSW, GSW, APA and Hope, p<0.01; increase in SC and LS predicted increase in GSW and APA, p<0.05 and increase in SC predicted increase in hope, p<0.05.*
**[29]**	Psychological wellbeing was significantly higher for those who exceeded MVPA guidelines, F= 10.90, *p*≤0.01, ηp^2 0.02 - ***small effect***									

Study	Psychological wellbeing (PW)	Self-worth (SW)	Self-esteem	Motivation towards physical activity	Hope	Depression	Anxiety	Emotional regulation	Other	*Any Identifiable biopsychosocial mechanisms and influencing factors*
**[63]**						PA was inversely associated with depression - +1hr of PA/wk = -11% odds of depression (OR=0.89, 95% CI 0.85-0.93, *p*=0.003) - ***strong association*** Longitudinal findings - no significant association between change in PA and odds of depression (OR=0.98, 95% CI 0.89-1.09, *p*=0.715) Direction of change was same as cross-sectional				**Gender was a moderator** - Girls had nearly twice the odds of depressive symptoms as boys (OR=1.82, 95% CI 1.49-2.24) Boys +1hr PA/wk = -8% odds of depression (OR = 0.92, 95% CI 0.87-0.97) - ***strong association*** Girls - higher PA = reduced odds of depression (OR= 0.92, 95% CI 0.85-1, *p*=0.044) - ***fairly strong association***
**[64]**			Self-esteem +9.04% following the intervention, M at T1 = 16.22±2.66, M at T2 = 17.68±2.16, *p*<0.01 - ***significant association***							Only girls included, so cannot be generalised across gender
**[65]**								Experimental group - Self-control skills (girls), significant +ve correlation with positive emotions (r=0.26, *p*<0.01) and		**Gender was a moderator** Significant direct link between changes in self-control skills and

Study	Psychological wellbeing (PW)	Self-worth (SW)	Self-esteem	Motivation towards physical activity	Hope	Depression	Anxiety	Emotional regulation	Other	*Any Identifiable biopsychosocial mechanisms and influencing factors*
								significant -ve association with negative emotions (r=0.16, *p*<0.01); hostility (r=0.12, *p*<0.05) and physical aggression (r=0.13, *p*<0.05) - ***Small effect sizes.*** Self-control skills (boys) - significant +ve correlation with positive emotions (r=0.23, *p*<0.01) and significant -ve association negative emotions (r=0.26, *p*<0.01); anger (r=0.22, *p*<0.01) and physical aggression (r=0.24, *p*<0.01) - ***Small effect sizes.*** Experimental vs control group: Self-control F=8.06, ηp^2=0.012, *p*<0.01); Negative emotions(F=17.04, ηp^2=0.026, *p*<0.001); Anger (F=19.52, ηp^2=0.029, *p*<0.001); Hostility (F= 17.02, ηp^2=0.026, *p*<0.001); Physical aggression (F=45.00, ηp^2=0.065, *p*<0.001); Teacher-rated behaviour (F=7.07, ηp^2=0.011, *p*<0.01) - ***Small effect sizes***		physical aggression in girls (β= -0.29, *p*=0.012) Boys higher self-control skills reduced hostility, mediated by increased +ve emotions and decreased -ve emotions (95% CI = -0.183, -0.063, *p*<0.001); significant direct link between hostility and physical aggression (β=0.62, *p*=0.003); Indirect link between changes in self-control and physical aggression (95% CI = -0.247, -0.045, *p*=0.004) As there was a direct link for girls but an indirect link for boys between self-control skills and aggression, the underlying mechanism may differ

Study	Psychological wellbeing (PW)	Self-worth (SW)	Self-esteem	Motivation towards physical activity	Hope	Depression	Anxiety	Emotional regulation	Other	*Any Identifiable biopsychosocial mechanisms and influencing factors*
**[62]**			Self-esteem control group - Mean difference T1-T2 = -0.30, *p*=0.895; Intervention group - Mean difference T1-T2 = 6.87, *p*=0.002; Score difference - 7.17, 95% CI 4.43-9.91, *p*<0.001. **Significant effect** *+2.3% intervention group score versus -0.1% control group score*			Depression control group - Mean difference T1-T2 = 0.02, *p*=0.968; Intervention group - Mean difference T1-T2 = -0.05, *p*=0.938; Score difference = -0.07, 95% CI - 0.83-0.69, *p*=0.436 **No significant effect on depression scores**	Anxiety Control group - Mean difference T1-T2 = -0.24, *p*=0.797; Intervention group - Mean difference T1-T2 = -1.18, *p*=0.049; Score difference = - 0.94, 95% CI - 1.43 to -0.45, *p*<0.001. **Significant effect** - *13.7% improvement in anxiety score of intervention group, compared to2.8% reduction for control*			Only adolescents were included. No separate data given for boys and girls.
**[61]**			Self-esteem: aerobic group - Mean difference T1-T2 = +2.0; control group - T1-T2 = +0.2 Significant aerobic group main effect F=9.35, *p*<0.01, ES=0.94, **Strong effect**			Significant difference in pre-test depression levels between the groups so analysis of covariance used Post-test: F1,63 = 6.93, *p*<0.05, ES = 0.74 **Significant decrease in aerobic group** depression levels **Moderate effect**	Significant difference in pre-test anxiety levels between the groups so analysis of covariance used - Post-test: F1,63= 0.40, *p*>0.05, ES=0.10 **No significant change.**			Cardiovascular fitness is a possible mechanism - CVF improved significantly within the aerobic group, compared to the control group. Suggests improved CVF = improved PW. But differing group baseline levels of anxiety and depression weaken the link

Study	Psychological wellbeing (PW)	Self-worth (SW)	Self-esteem	Motivation towards physical activity	Hope	Depression	Anxiety	Emotional regulation	Other	*Any Identifiable biopsychosocial mechanisms and influencing factors*
**[31]**									Total mood disturbance (TMD) = included scores for: anger, confusion and depression. **No significant TMD differences between Box'Tag and Rock+Water groups at any time** T1 - F=0.61, *p*=0.44; T2 - F=0.22, *p*=0.88; T3 - F=0.11, *p*=0.74; T4 - F=0.16, p=0.74 Total difficulties (TDS) = scores for: emotional regulation and peer relationship problems. **No significant TDS differences between groups at any time**: T1 - F=0.06, *p*=0.80; T2 - F=0.31, *p*=0.58; T3 - F=0.41, *p*=0.53; T4 - F=0.34, *p*=0.56 **No significant effect at all**	Age may be a moderator on overall mood as all children were aged 11 to 12 and may be undergoing puberty.
**[66]**								Initial level of self-control was predicted		

Study	Psychological wellbeing (PW)	Self-worth (SW)	Self-esteem	Motivation towards physical activity	Hope	Depression	Anxiety	Emotional regulation	Other	*Any Identifiable biopsychosocial mechanisms and influencing factors*
								by social competence in sport r=0.26, social responsibility r=0.14, *small correlations* and teamwork r= 0.53, *moderate correlation* (*p*<0.05 for all) The small increase in self-control was predicted by teamwork r=-0.51 and a sense of belonging r=0.54, *moderate correlations* (*p*<0.05 for all). **No significant change in self-control**		
**[38]**	Psychological wellbeing was positively correlated with PA level, F=12.70, $p<0.001$, $\eta^2 = 0.030$. When controlling for other variables, PW was still higher for participants who reported higher PA levels, F=6.79, $p<0.001$, $\eta^2 = 0.016$ **Small effect size**									**Volume of PA was a moderator** Higher PA levels led to an increase in all HRQoL measures, irrespective of age, gender, BMI, SES and CRF. However, the effect size was small for all.
**[35]**	The intervention was found to									**Changes in potential mediators correlated with**

Study	Psychological wellbeing (PW)	Self-worth (SW)	Self-esteem	Motivation towards physical activity	Hope	Depression	Anxiety	Emotional regulation	Other	*Any Identifiable biopsychosocial mechanisms and influencing factors*
	enhance overall psychological wellbeing, d=0.35, *p*<0.05 **Small but significant effect**									**changes in wellbeing:** autonomy choice (A=0.15, SE=0.06, *p*=0.016); screen time (A=-0.21, SE=0.06, *p*<0.001); muscular fitness (A=0.012, SE=0.05, *p*=0.011); resistance training skill competency (A=0.48, SE=0.04, *p*<0.001). Multiple factors were involved so a causal link between enhanced PA and improved autonomy cannot be applied
**[57]**						**Attendance effect** - there was a significant decrease in depression for regular attendees, but not for those who attended infrequently. **Age Effect** - elementary school intervention participants showed a decrease in depression, the control group did not. No	Anxiety decreased significantly for those in the yoga intervention group, but not for the control group F=3.87, *p*<0.05, d=0.21 **Small but significant effect**	**A significant effect was found for aggression** F=36.61, *p*<0.05. The level of change was found to relate to the initial levels of aggression. Children in the yoga group with higher initial aggression levels showed a greater reduction in aggression, than those with lower initial levels		**Age was a moderator** - prosocial behaviour improved in adolescents only, whereas depression decreased in the elementary school children only **Gender was a moderator** – Depression decreased in boys only

Study	Psychological wellbeing (PW)	Self-worth (SW)	Self-esteem	Motivation towards physical activity	Hope	Depression	Anxiety	Emotional regulation	Other	*Any Identifiable biopsychosocial mechanisms and influencing factors*
						differences for the adolescents. **Gender effect** - The decrease in depression for boys in the intervention was significant, but not for girls. **No significant main effect**				
**[32]**								Primary coping (F=12.39, *p*=0.02, d=0.15), and emotional regulation (F=4.90, *p*=0.05, d=0.12) increased post-intervention. Secondary coping (F=9.75, *p*=0.01, d=0.14) and positive thinking (F=5.75, *p*=0.05, d=0.13)also improved **Small effect size** No significant effect for attitudes towards violence (F=4.00, *p*=0.09, d=-0.29)		Adolescents only. No separate analysis completed for girls and boys.
**[67]**		GSW did not differ significantly between the 2 groups, pre- or post-intervention						Post intervention the yoga participants showed a **significant reduction in negative behaviours**, in contrast to the non-yoga group		

Table S3. – Results for social and physical mental health and wellbeing outcomes.

Study	Social – Social competence (SC)	Social – Social responsibility (SR)	Social – Teamwork	Social – Support from others	Physical – Physical wellbeing	Physical – Physical competence (PC)	Physical – Physical self-worth (PSW)	Any Identifiable biopsychosocial mechanisms and influencing factors
[30]	**Social competence - significant increase overall,** F =15.67, η^2 =0.07, p<0.01. When split by gender ***only girls result was significant*** F=12.78, η^2 =0.11, p<0.01 - ***moderate effect*** T2 - *Moderate + correlation with PC (r=0.62), PSW (r=0.58), SW (r=0.52), APA (r=0.64) and hope (r=0.45)*			**Leader Support (LS) - No significant change overall.** *Age was a moderator* - LS: ***9-11 yrs group, significant decrease,*** F=2.64, η^2 = 0.03, p<0.05 but no change for 12-16 yrs group, - ***small effect*** *T2 - Moderate + correlation with hope (r=0.53)*		**Physical competence (PC) - significant increase overall,** F=18.41, η^2 = 0.09, p<0.01 - ***small effect*** *T1 - Moderate +Correlation with social competence (r=0.43) T2 - Strong + correlation with PSW (r=0.72) and APA (r=0.73) Moderate +correlation with SW (r=0.62) and hope (r=0.44)*	**PSW - significant increase overall**, F=11.12, η^2 = 0.05, p<0.01 - ***small effect*** Age was a moderator - **12-16 yrs- significant increase**, no change for 9-11 yrs, F=17.26, η^2 = 0.14, p<0.01 - ***moderate effect*** *T1 - Moderate +correlation with PC (r=0.62) and SC (r=0.44) T2 - Strong + correlation with SW (r=0.72) and APA (r=0.73) Moderate + correlation with hope (r=0.46)*	**Age was a moderator** - LS: 9-11 yrs, significant decrease but no change for 12-16 yrs; PSW and APA: 12-16 yrs, significant increase, no change for 9-11 yrs. **Gender was a moderator** - SC: only girls showed a significant increase. Increase in PC predicted increase in PSW, GSW, APA and Hope, p<0.01; increase in SC and LS predicted increase in GSW and APA, p<0.05 and increase in SC predicted increase in hope, p<0.05.
**[29]**	**Parental and autonomy support was significantly higher in children with higher MVPA**, F= 6.10, p=0.01, ηp^2 0.11 - ***medium effect*** and social support, F=4.60, $p \leq 0.03$, ηp^2 0.01 - ***small effect***				**Physical wellbeing was significantly higher for children with higher MVPA** F=31.77, $p \leq 0.01$, ηp^2 0.06 - ***small effect***			**Gender was a moderator** - The results for girls were significantly higher than the boys in autonomy and parent relations and social support and peers, leading to a greater total wellbeing score for girls.
**[66]**	Initial level of SC was predicted by pre-camp self-control r=0.31 - *small correlation*, social responsibility r=0.11 -	**SR increased significantly at group level.** Initial level of SR was predicted by age r=-	Initial level of teamwork was predicted by SC r=0.40, self-control r=0.44, *moderate*			Initial level of Perceived sport competence was predicted by effort r=0.41, p<0.05,		Although social responsibility was the only outcome which increased significantly post-camp, an enhanced

	small correlation, and teamwork r=0.50 - *moderate correlation* (*p*<0.05 for all). The small increase in SC was predicted by a significant increase in teamwork r=-0.46, *p*<0.05 and a sense of belonging r=0.56, *p*<0.05 - *Moderate correlations* **No significant increase in SC**	0.20, SC r=0.23, self-control r=0.23 and effort r=0.23 (*p*<0.05 for all) - *small correlations* The change in SR was predicted by age r=0.14, *p*<0.05, *small correlation*, and sense of belonging r=0.43, *p*<0.05, *moderate correlation* **Significant increase in SR, r =0.41, *p*<0.05 - *Moderate effect***	*correlations*, and effort r=0.19, *small correlation* (*p*<0.05). The teamwork change was predicted by SC r - 0.32, self-control r=0.24, *small correlations*, and sense of belonging r=-0.64 *moderate correlation* (*p*<0.05) **No significant change in teamwork**			*moderate correlation* The small increase in PC was predicted by a significant increase in effort r=-0.32 and a sense of belonging r=0.37 (*p*<0.05 for all), *small correlations* **No significant change in PC**		sense of belonging predicted change in all outcomes.
**[38]**				***Autonomy and parent relations was positively correlated with PA,*** F=15.90, *p*<0.001, η^2 = 0.037. Controlling for other variables, the level was still higher for participants who reported higher PA , F=12.11, *p*<0.001, η^2 = 0.029. ***Small effect*** *Peer support +ve correlation with PA*, F=12.74, *p*<0.001, η^2 = 0.030. Controlling for other variables, the level was still higher for participants who reported higher PA, F=6.56, *p*<0.001, η^2 = 0.016 ***Small effect***	***Physical wellbeing level was positively correlated with PA level***, F=20.52, *p*<0.001, η^2 = 0.047. When controlling for other variables, physical wellbeing was still higher for participants who reported higher PA levels, F=15.89, *p*<0.001, η^2 = 0.037. ***Small effect size.***			**Volume of PA was a moderator** Higher PA levels led to an increase in all HRQoL measures, irrespective of age, gender, BMI, SES and CRF. *However, the effect size was small for all.*
**[35]**				Level of autonomy choice increased A=-0.21, SE=0.6, p=0.16 and *autonomy choice was a mediator for improved wellbeing* AB=0.026, 95% CI =				***Changes in potential mediators correlated with changes in wellbeing: autonomy choice (A=0.15, SE=0.06, p=0.016); screen time (A=-0.21, SE=0.06,***

		0.004-0.055 *Small effect*	***p<0.001); muscular fitness (A=0.012, SE=0.05, p=0.011); resistance training skill competency (A=0.48, SE=0.04, p<0.001).*** The intervention involved multiple factors, not just enhanced PA, therefore it is difficult to apply a causal link between the enhanced PA and improved autonomy, and subsequently enhanced wellbeing
**[57]**	The intervention positively impacted peer-reported pro-social behaviour, but only in high school students and this level of pro-social behaviour was maintained following the programme, whereas pro-social behaviour decreased in the younger children post-programme.		**Age was a moderator** for both peer-reported pro-social behaviour and depression, but prosocial behaviour improved in the high school children only, whereas depression decreased in the elementary school children only **Gender was a moderator** - only boys in the intervention group showed a reduction in depression
